# Dermatologic Conditions and Incident Anxiety in Young Adults: Propensity Score–Matched Retrospective Cohort Study

**DOI:** 10.2196/90820

**Published:** 2026-06-03

**Authors:** Isabella Zai, Adrian Zai

**Affiliations:** 1 Westwood High School, Westwood, MA, United States; 2 Population and Quantitative Health Sciences, University of Massachusetts Chan Medical School, 368 Plantation Street, Worcester, MA, 01605, United States, 1 617-256-4120

**Keywords:** dermatologic diseases, anxiety disorders, young adult, electronic health records, propensity score matching

## Abstract

**Background:**

Dermatologic conditions such as acne, psoriasis, and dermatitis commonly affect young adults and may contribute to psychological distress. While prior studies have suggested an association between skin disease and anxiety, longitudinal population-level evidence in young adults remains limited.

**Objective:**

This study aimed to examine the association between common dermatologic conditions and the incidence of anxiety among young adults using a large electronic health record–based cohort.

**Methods:**

We conducted a retrospective cohort study using the TriNetX Research Network, including young adults aged 18 to 22 years with and without a qualifying dermatologic diagnosis between 2019 and 2020. The index date was defined as the first dermatologic diagnosis for exposed individuals and a qualifying ambulatory visit for controls. Individuals with a prior diagnosis of anxiety were excluded. Propensity score matching was used to balance demographic characteristics and ambulatory visit history between cohorts. Incident anxiety diagnoses were assessed at 1, 3, and 5 years following the index date. Cumulative incidence, absolute risk differences, risk ratios, and time-to-event analyses were evaluated.

**Results:**

After propensity score matching, 169,720 individuals were included in each cohort. Young adults with dermatologic conditions consistently exhibited a higher incidence of anxiety across all time points. At 1 year, anxiety occurred in 3.9% (5838/149,464) of individuals in the dermatologic condition group compared with 3.4% (3237/95,020) of controls. By 3 years, incidence increased to 11.8% (17,559/149,464) of individuals vs 10.4% (9906/95,020) of controls, and by 5 years to 16.8% (25,184/149,464) of individuals vs 15.5% (14,712/95,020) of controls. Absolute risk differences widened over time, from 0.50 to 1.40 percentage points. Time-to-event analyses demonstrated a modest but consistent increase in hazard, with hazard ratios ranging from 1.12 to 1.14 (all *P*<.001).

**Conclusions:**

In this large, propensity score–matched cohort of young adults, common dermatologic conditions were associated with a small but persistent increase in the incidence of anxiety over time. Although absolute differences were modest, the consistency of findings across multiple analytic approaches highlights the importance of considering psychological well-being as part of comprehensive care for young adults with dermatologic conditions.

## Introduction

Dermatologic conditions are among the most common reasons for ambulatory health care visits in adolescents and young adults [1,2]. Disorders such as acne, psoriasis, and dermatitis frequently emerge or worsen during late adolescence and early adulthood, a developmental period characterized by significant psychological, social, and identity-related transitions [3-6]. Although the physical manifestations of skin disease are readily apparent, their potential mental health sequelae are less consistently recognized in routine clinical care [7-13].

Prior studies have suggested an association between dermatologic conditions and adverse mental health outcomes, including anxiety [7,14,15]. However, much of this literature relies on cross-sectional surveys, clinic-based samples, or patient-reported measures of psychological distress [15-19]. While these approaches provide important insights into symptom burden and quality of life, they are limited in their ability to establish temporal relationships or estimate population-level risk [20,21]. In addition, many studies focus on adults or pediatric populations, with comparatively less attention to young adults navigating the transition between adolescent and adult care [5,22-25].

Electronic health record (EHR) data offer an opportunity to examine longitudinal associations between dermatologic diagnoses and subsequent mental health outcomes at scale [26-28]. Large, multi-institutional EHR networks allow for the identification of incident diagnoses, the alignment of outcomes to clinically meaningful index events, and the evaluation of risk over extended follow-up periods [29-31]. Such data can complement survey-based and qualitative work by providing estimates of real-world diagnostic patterns and temporal trends across diverse care settings.

Despite increasing interest in psychodermatology, few studies have leveraged longitudinal EHR data to quantify the risk of incident anxiety following dermatologic diagnoses in young adults. In particular, there is limited evidence characterizing how anxiety diagnoses accumulate over time after an initial dermatologic diagnosis, or how this risk compares with peers without dermatologic conditions when baseline differences in demographics and health care use are accounted for.

The objective of this study was to evaluate the association between common dermatologic diagnoses and subsequent incident anxiety among young adults aged 18 to 22 years using a large federated EHR network. We conducted a propensity score–matched cohort study comparing cumulative incidence and time to anxiety diagnosis at 1, 3, and 5 years of follow-up. By focusing on incident anxiety and aligning outcomes to clearly defined index events, this study aimed to provide population-level estimates of anxiety risk following dermatologic diagnoses during a critical developmental period.

## Methods

### Study Design and Data Source

We conducted a retrospective matched cohort study using deidentified EHR data accessed through the TriNetX Research Network, a federated platform comprising multiple health care organizations across the United States [32]. The network contains longitudinal inpatient and outpatient data, including demographics, diagnoses, and encounter information. All analyses were performed within the secure analytic environment provided by TriNetX using deidentified data in accordance with institutional policies.

The study was reviewed by the institutional review board and determined to be exempt.

### Study Population

The study population included young adults aged 18 to 22 years with clinical encounters recorded between January 1, 2019, and December 31, 2020. The 2019 to 2020 index period was selected to allow sufficient longitudinal follow-up of up to 5 years within the available TriNetX data while capturing a contemporary cohort of young adults receiving routine care. This period was not selected to coincide with the COVID-19 pandemic but rather reflects the most recent interval permitting extended follow-up. To ensure engagement with the health care system, patients were required to have at least one qualifying ambulatory visit during the study period.

Patients were followed longitudinally for the development of incident anxiety diagnoses through December 31, 2025, allowing for up to 5 years of potential follow-up.

### Exposure Definition and Index Date

The exposed cohort consisted of patients with a diagnosis of a dermatologic condition, defined by the presence of *International Classification of Diseases, Tenth Revision* (*ICD-10*) diagnosis codes corresponding to common dermatologic disorders. The index date for exposed patients was defined as the date of the first qualifying dermatologic diagnosis during the study period.

The comparison cohort consisted of patients without any recorded dermatologic diagnosis at any time prior to or during the study period. For these patients, the index date was defined as the date of the first qualifying ambulatory visit occurring within the same calendar period.

### Outcome Definition

The primary outcome was incident anxiety, defined as a new diagnosis of an anxiety disorder recorded after the index date, identified using *ICD-10* diagnosis codes for anxiety disorders.

To focus on incident anxiety, patients with any recorded anxiety diagnosis during the 1 year before the index date were excluded at the cohort definition stage. In addition, outcome analyses excluded patients with anxiety diagnoses occurring prior to the start of each analytic time window to ensure that all patients included in the risk set were at risk for incident anxiety during the specified follow-up period.

Outcome time windows began 1 day after the index date to reduce misclassification of anxiety diagnoses recorded during the same clinical encounter as the dermatologic diagnosis or index visit.

### Covariates

Baseline covariates included age, sex, race, and ethnicity, as well as measures of health care use prior to the index date, including the presence of ambulatory visits. Covariates were selected a priori based on clinical relevance and their potential role as confounders of the association between dermatologic diagnoses and subsequent anxiety.

Anxiety diagnoses were not included as matching covariates because patients with prior anxiety were excluded from the study population, and postindex variables were not incorporated into propensity score estimation.

### Propensity Score Matching

To reduce confounding, propensity score matching was performed to create balanced cohorts of patients with and without dermatologic diagnoses. Propensity scores were estimated using logistic regression based on baseline demographic characteristics and health care use measures.

Patients were matched one-to-one using nearest neighbor matching without replacement. Balance between cohorts was assessed using standardized mean differences, with values less than 0.1 indicating acceptable balance.

All outcome analyses were conducted using the matched cohorts.

### Follow-Up and Time Horizons

Patients were followed from the index date until the earliest of the following events: first recorded anxiety diagnosis, end of the specified follow-up window, or last available encounter in the dataset.

Separate analyses were conducted using follow-up windows of 1, 3, and 5 years following the index date. As follow-up duration increased, fewer patients contributed data to later time horizons due to censoring at the end of available follow-up.

### Statistical Analysis

Cumulative incidence of anxiety was calculated for each cohort at 1, 3, and 5 years following the index date. Absolute risk differences and relative risks with 95% CIs were reported.

Time to incident anxiety was additionally evaluated using Kaplan-Meier methods, with differences between cohorts assessed using the log-rank test. Hazard ratios with 95% CIs were estimated to quantify relative differences in the rate of anxiety diagnosis over time. Kaplan-Meier analyses were performed as supportive time-to-event analyses to assess consistency with cumulative incidence findings.

All statistical tests were 2-sided, and statistical significance was defined as *P*<.05.

### Sensitivity Analyses

Sensitivity analyses were conducted by comparing effect estimates across multiple follow-up horizons to assess the robustness and temporal consistency of findings.

### Ethical Considerations

This study was reviewed by the Institutional Review Board of UMass Chan Medical School and determined to be exempt from human subjects research (STUDY00002783). The research used deidentified EHR data accessed through the TriNetX Research Network. Because the data were deidentified prior to analysis and posed minimal risk to participants, no direct identifiers were available to the investigators, participants were not contacted, and no attempts were made to reidentify individuals, informed consent was not required in accordance with the Common Rule provision for exempt secondary research [33,34] and the HIPAA (Health Insurance Portability and Accountability Act) Privacy Rule standard for deidentification.

Data were analyzed within the secure TriNetX analytics environment, and results were reported only in aggregate form. No participant compensation was provided, as this study did not involve direct participant contact.

## Results

### Cohort Assembly and Baseline Characteristics

**Figure 1.**
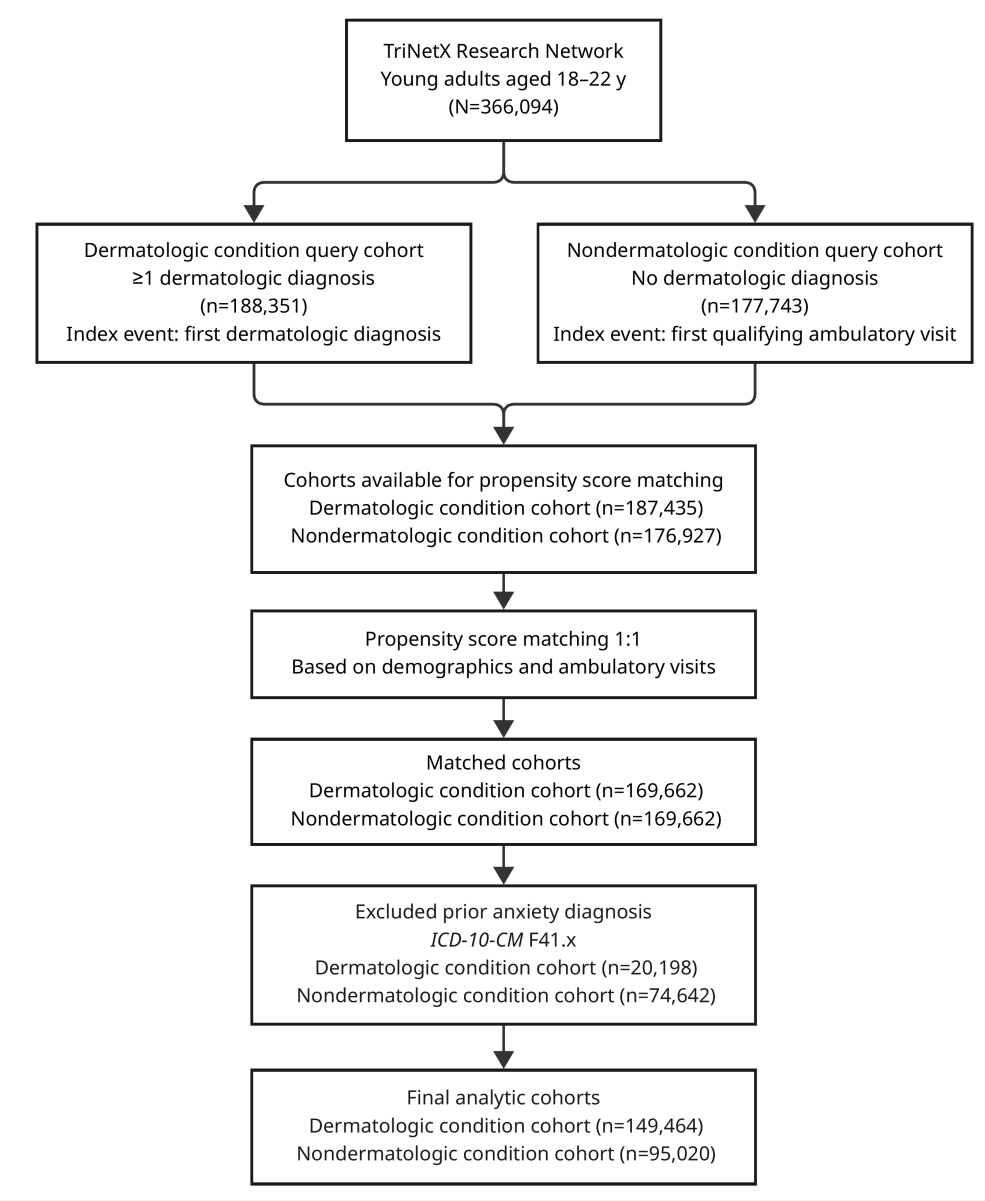
Cohort selection flow diagram. *ICD-10-CM*: *International Classification of Diseases, Tenth Revision, Clinical Modification*.

Figure 1 summarizes cohort assembly. Among young adults aged 18 to 22 years with a qualifying dermatologic diagnosis between January 1, 2019, and December 31, 2020, eligible individuals were matched 1:1 to controls without dermatologic diagnoses using propensity scores. After matching, 169,720 individuals were retained in each cohort.

Baseline demographic characteristics before and after matching are shown in Table 1. Prior to matching, modest differences were observed across race, ethnicity, sex, and ambulatory visit history. After propensity score matching, covariates were well balanced between cohorts, with standardized mean differences below 0.01 for all matched variables.

**Table 1.** Baseline characteristics of propensity score–matched young adults with and without dermatologic diagnoses^a^.

Characteristic	Dermatology cohort (n=169,662), n (%)	Nondermatology cohort (n=169,662), n (%)	Standardized mean difference
Sex			
Female	96,440 (56.8)	96,257 (56.7)	0.0018
Male	73,139 (43.1)	73,325 (43.2)	0.0019
Race and ethnicity			
American Indian or Alaska Native	1018 (0.6)	10,542 (0.6)	0.0024
Asian	9289 (5.5)	9356 (5.5)	0.0017
Black or African American	27,461 (16.2)	27,451 (16.2)	0.0001
Hispanic or Latino	23,930 (14.1)	23,610 (13.9)	0.0052
Native Hawaiian or Other Pacific Islander	886 (0.5)	9198 (0.5)	0.0026
Not Hispanic or Latino	112,308 (66.2)	112,696 (66.4)	0.0047
White	107,599 (63.4)	107,415 (63.3)	0.0023
Prior ambulatory visit before index	98,656 (58.1)	98,495 (58.1)	0.0019

a Cohorts were matched 1:1 using propensity score matching based on sex, race, ethnicity, and prior ambulatory health care use. All standardized mean differences were less than 0.01, indicating excellent postmatching balance.

Young adults aged 18 to 22 years were identified from the TriNetX Research Network between 2019 and 2020. Patients were categorized based on the presence or absence of a qualifying dermatologic diagnosis (including acne, psoriasis, or dermatitis or eczema), and those with a prior diagnosis of anxiety were excluded. Propensity score matching was applied to create balanced cohorts with and without dermatologic conditions. The final matched cohorts were followed for incident anxiety diagnoses at 1-, 3-, and 5-year intervals after the index date.

### Incident Anxiety at 1, 3, and 5 Years

Table 2 presents the cumulative incidence of newly diagnosed anxiety disorders at 1, 3, and 5 years following the index date. Across all time horizons, young adults with dermatologic conditions experienced a consistently higher incidence of anxiety compared with matched controls. The absolute difference between cohorts increased over time, while the relative increase in risk remained modest across follow-up periods.

**Figure 2.**
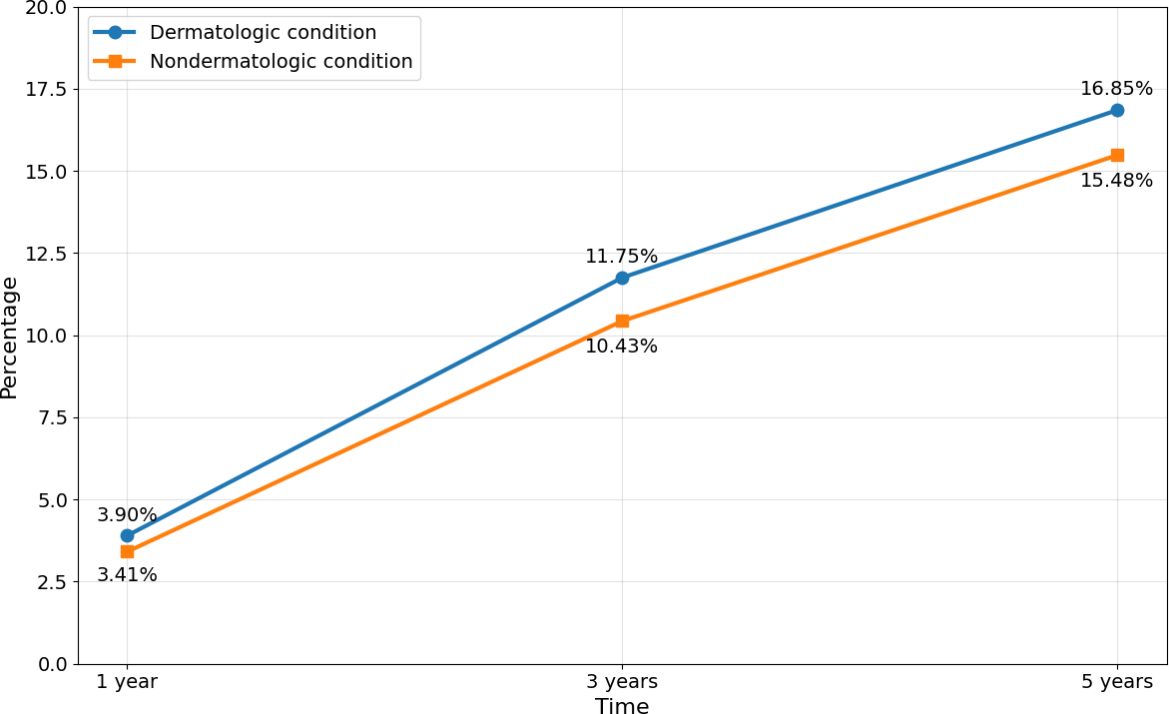
Cumulative incidence of anxiety among young adults with and without dermatologic conditions. Cumulative incidence of new-onset anxiety at 1, 3, and 5 years after the index date among propensity score–matched young adults aged 18 to 22 years with dermatologic conditions diagnosed between 2019 and 2020 and matched peers without dermatologic conditions. Percentages represent the proportion of individuals with a new anxiety diagnosis within each follow-up interval, excluding those with anxiety prior to the index date.

Figure 2 illustrates these cumulative incidence estimates over time, demonstrating a parallel increase in anxiety diagnoses in both cohorts with a persistently higher absolute risk among individuals with dermatologic conditions.

**Table 2.** Risk of incident anxiety among young adults with vs without dermatologic conditions^a^.

Follow-up period and cohort	Incident anxiety, n (%)	Risk difference (%; 95% CI)	Risk ratio (95% CI)	Odds ratio (95% CI)
1 year				
With a dermatologic condition (n=149,464)	5838 (3.9)	0.50 (0.30‐0.70)	1.147 (1.099‐1.196)	1.153 (1.103‐1.204)
Without a dermatologic condition (n=95,020)	3237 (3.4)	Reference	Reference	Reference
3 years				
With a dermatologic condition (n=149,464)	17,559 (11.8)	1.32 (1.07‐1.58)	1.127 (1.101‐1.153)	1.144 (1.114‐1.174)
Without a dermatologic condition (n=95,020)	9906 (10.4)	Reference	Reference	Reference
5 years				
With a dermatologic condition (n=149,464)	25,184 (16.8)	1.40 (1.10‐1.70)	1.088 (1.068‐1.109)	1.106 (1.082‐1.131)
Without a dermatologic condition (n=95,020)	14,712 (15.5)	Reference	Reference	Reference

a Patients with a prior diagnosis of anxiety before the index date were excluded. Analyses were conducted after propensity score matching.

### Time-to-Event Analysis

Time-to-event analyses using Kaplan-Meier methods further supported these findings. Individuals with dermatologic conditions exhibited a modestly increased hazard of incident anxiety across follow-up periods.

The hazard ratio for incident anxiety was 1.135 (95% CI 1.088‐1.185) at 1 year, 1.121 (95% CI 1.094‐1.149) at 3 years, and 1.096 (95% CI 1.074-1.119) at 5 years, consistent with the cumulative incidence findings in Table 2; log-rank tests were significant across all analyses (all *P*<.001).

Despite statistical significance, Kaplan-Meier curves showed substantial overlap between cohorts, reflecting the modest magnitude of the association and reinforcing the importance of interpreting relative measures alongside absolute risk differences.

## Discussion

In this large, propensity score–matched cohort study of young adults, we found that individuals with common dermatologic conditions experienced a consistently higher incidence of anxiety compared with matched peers without dermatologic conditions. Although the magnitude of the association was modest, the excess risk was observed across several follow-up intervals and persisted over time. These findings suggest that dermatologic conditions in early adulthood are associated with a small but sustained increase in the likelihood of subsequent anxiety diagnoses. The consistency of findings across cumulative incidence, relative risk, and time-to-event analyses supports the robustness of the observed association.

The absolute differences in anxiety incidence were relatively small, ranging from approximately one-half of a percentage point at 1 year to just over 1 percentage point at 5 years. However, given the high prevalence of dermatologic conditions among young adults, even modest increases in risk may have meaningful implications at the population level. Importantly, the consistency of the association across 1-, 3-, and 5-year follow-up intervals strengthens confidence that the observed relationship is not driven by short-term diagnostic clustering or surveillance bias alone.

The relative effect size attenuated with longer follow-up, while the absolute difference in cumulative incidence increased over time. This pattern is expected in large observational cohorts and reflects the increasing background incidence of anxiety in young adulthood. From a clinical perspective, the absolute risk difference may be more informative than relative measures, as it better contextualizes the magnitude of excess risk attributable to dermatologic conditions. Our findings underscore the importance of interpreting statistically significant associations in the context of absolute effect sizes.

Several mechanisms may plausibly explain the observed association. Dermatologic conditions, such as acne, psoriasis, and dermatitis, often emerge or worsen during adolescence and early adulthood, a period characterized by heightened psychosocial vulnerability. Visible skin disease may contribute to anxiety through effects on self-image, social interactions, and perceived stigma. In addition, chronic or recurrent symptoms, such as pruritus, pain, or flares, may increase psychological distress over time. While our study was not designed to identify causal pathways, the persistence of the association across multiple years is consistent with a cumulative psychosocial burden rather than a transient response to diagnosis alone.

Our findings are consistent with prior literature reporting associations between dermatologic disease and mental health outcomes, while extending this work in several important ways. First, we focused specifically on young adults, a population in which both dermatologic conditions and anxiety commonly emerge but remain underrepresented in large-scale longitudinal studies. Second, by using propensity score matching and excluding individuals with prior anxiety diagnoses, we sought to minimize confounding and focus on incident outcomes. Third, the use of multiple follow-up intervals allowed us to characterize the temporal evolution of risk rather than relying on a single time point estimate.

This study has several limitations. As an observational analysis based on EHR data, it is subject to residual confounding despite propensity score matching. The study period included the onset of the COVID-19 pandemic, which may have influenced background rates of anxiety diagnoses as well as patterns of health care use. Although both cohorts were drawn from the same calendar period and matched on baseline characteristics and ambulatory visit history, residual confounding related to pandemic-era behavioral and health system changes remains possible. However, because both cohorts were derived from the same period, such effects are likely to be nondifferential and unlikely to materially affect the internal validity of the comparison. Anxiety diagnoses may be underascertained or misclassified, and diagnostic practices may vary across health care organizations. Additionally, patients with dermatologic conditions may have more frequent health care encounters, potentially increasing opportunities for anxiety diagnosis. Although matching on ambulatory visit history partially addresses this concern, differential health care use cannot be fully excluded. Finally, the TriNetX platform does not allow for detailed assessment of disease severity, symptom burden, or patient-reported outcomes, which may further modify the relationship between dermatologic conditions and anxiety.

Despite these limitations, the strengths of this study include its large, geographically diverse population, rigorous matching strategy, and consistent findings across multiple analytic approaches. The concordance between cumulative incidence estimates and time-to-event analyses supports the robustness of the observed association.

In conclusion, young adults with common dermatologic conditions experience a small but persistent increase in the incidence of anxiety compared with matched peers without dermatologic conditions. These findings highlight the importance of holistic care approaches that consider both physical and psychological well-being in patients with dermatologic disease. Future studies incorporating patient-reported outcomes and disease severity measures may help clarify which subgroups are at greatest risk and inform targeted screening or intervention strategies.

## Abbreviations

EHR: electronic health record
HIPAA: Health Insurance Portability and Accountability Act
*ICD-10*: *International Classification of Diseases, Tenth Revision*
